# The Panel Doctors' Decision

**Published:** 1923-12

**Authors:** 


### THE PANEL DOCTORS' DECISION.

THE decision of the panel doctors to accept the terms of the Minister's final offer is not the last word in an unhappy controversy. The question of the proper capitation fee is, by reason of that acceptance, to be submitted to an independent Court of Inquiry, before which all parties to the dispute will be heard. And so, for some time to come, the matter of the remuneration of the medical man for his services will be dragged up and down before the gaze of the public, who are getting a little bit weary of it.

### A Ridiculous Performance.

But the decision to accept the findings of a Court of Inquiry rather than the Minister's alternative offer of 8s. 6d. will have the very considerable advantage, from the point of view of the Minister, that he will have a complete answer to the contention of the approved societies that he is recklessly throwing away the funds which belong to the insured persons. It is impossible to take very seriously the phrases to which the society leaders have committed themselves, such as "sheer spoliation" and "betrayal of trust," or their ridiculous performance in sending a wireless message to Mr. Lloyd George on the *Majestic* to come home and save the "Good old Insurance Act."

### What Do the Societies Mean?

If it is the contention of the societies—and with the best will in the world, frankly we find it difficult to know what they are driving at—that the sum proposed by the Minister to be paid to the doctors, 8s. 6d., is, on merits, excessive, the Court of Inquiry will set that at rest. If, on the other hand, the contention is that any amount for medical benefit in excess of that provided for in the original actuarial estimates is an improper charge on Insurance funds, we say that Parliament and the public will not hear of any such charge being thrown on the Exchequer. The Minister has said that he is advised by the Government Actuary that he can pay the 8s. 6d. for a term of years without imperilling either the normal benefits or the continuance of additional benefits. And yet the societies—many of them in a position, owing to their extremely favourable sickness experience, to give additional benefits altogether in excess of those contemplated by the small actuarial margin in the original estimates of 1911-12—are allowing themselves the luxury of the language we have quoted.

### The Separate Unit Bogey.

In a wholly superfluous letter of probably 10,000 words with which the "Emergency Committee of the Provisional General Council of Approved Societies" have thought fit to bombard the unfortunate Sir Wm. Joynson-Hicks, there are statements, bearing upon a particular side of the controversy, which are deserving of a separate comment. The societies say: "It is possible that in the mass (that is, in general) approved societies might expect to possess at the second valuation a surplus which—after providing for the present continuance of their existing rates or schemes of additional benefits—might leave them with sufficient to pay a capitation fee of 8s. 6d. to the doctors for a limited period of time, if it were proper to utilise their members' surplus for that purpose. Societies, however, are not valued in the mass, but as individual and independent financial units, and it is incontrovertible that every society would not be in such a comparatively favourable position." And again: "It may be confidently anticipated that, at the second valuation, certain societies will be quite unable to meet any further general charge on their funds such as is now contemplated by the Minister." With regard to these statements, it may be said at once that there is a special fund, the Central Fund, of a very considerable amount, available for spreading the risks between societies to enable some of the less fortunate to pay their way, and especially to help these societies to meet the special loss of falling contributions through unemployment. Further, a system which required that many or most societies should have excessive funds (provided not only by the insured persons, but by their employers and the State) in order that others, similarly financed, should be able to pay their way would stand self-condemned. So much for the value of the "separate unit" argument.

### The Insured Person's Point of View.

Finally, when on merits, to which alone the Court of Inquiry will address themselves, it is decided that a certain amount is the appropriate capitation fee, the public will not be slow to appreciate that, unless the sum is charged wholly against existing Insurance funds, some part must be found from new contributions or by the taxpayer. We have no doubt that they will see that the Exchequer is not raided. The insured persons, being left with the alternative of paying a new contribution or carrying on as at present, would obviously, if they had any means of expressing themselves collectively, unhesitatingly choose the latter course, and doubtless at the same time express themselves pretty vigorously on the subject of camouflage.

